# Adolescents’ discourse on the consumption of psychoactive substances in schools

**DOI:** 10.1590/0034-7167-2024-0269

**Published:** 2025-11-07

**Authors:** Karla Nataly Ferreira Vasconcelos, Helder de Pádua Lima, Nathan Aratani, Bianca Cristina Ciccone Giacon-Arruda, Soraia Geraldo Rozza, Sarah Lima Verde da Silva, Eysler Gonçalves Maia Brasil, Flavia Milene de Souza Nobre

**Affiliations:** I Fundação Universidade Federal de Mato Grosso do Sul. Coxim, Mato Grosso do Sul, Brazil; II Secretaria da Saúde do Ceará. Fortaleza, Ceará, Brazil; III Universidade da Integração Nacional da Lusofonia Afro-Brazileira. Redenção, Ceará, Brazil; IV Faculdade Estácio de Alagoinhas. Alagoinhas, Bahia, Brazil

**Keywords:** Health Promotion, Mental Health, Adolescent, Adolescent Behavior, Psychotropic Drugs., Promoción de la Salud, Salud Mental, Adolescente, Percepción, Psicotrópicos.

## Abstract

**Objectives:**

to understand the perception of adolescents in public schools regarding the use of psychoactive substances.

**Methods:**

a qualitative exploratory study, carried out at a public school with 14 adolescents. Data were collected through a self-administered sociodemographic questionnaire and focus group, in which a semi-structured script of questions was used, organized according to the Discourse of the Collective Subject.

**Results:**

adolescents’ perceptions about the use of psychoactive substances revealed judgments, doubts and knowledge gaps. Participants’ daily lives were marked by greater acceptance, easy access and consumption of legal chemical substances in the territory, including in school environments.

**Final Considerations:**

it is necessary to enhance school environments with participatory, horizontal and intersectoral actions for prevention and health promotion that include students, families and the school, aiming at reflection on drug use and lifestyles, and the redefinition of concepts and perceptions.

## RESUMEN

**Objetivos:**

comprender la percepción de los adolescentes de las escuelas públicas sobre el uso de sustancias psicoactivas.

**Métodos:**

estudio exploratorio cualitativo, realizado en una escuela pública con 14 adolescentes. Los datos fueron recolectados a través de un cuestionario sociodemográfico autoadministrado y de un grupo focal, en el que se utilizó un guion de preguntas semiestructurado, organizado según el Discurso del Sujeto Colectivo.

**Resultados:**

las percepciones de los adolescentes sobre el uso de sustancias psicoactivas revelaron juicios, dudas y lagunas en el conocimiento. La vida cotidiana de los participantes se evidenció permeada por una mayor aceptación, fácil acceso y consumo de sustancias químicas legales en el territorio, incluso en las escuelas.

**Consideraciones Finales:**

es necesario mejorar el ambiente escolar con acciones participativas, horizontales e intersectoriales de prevención y promoción de la salud que incluyan a los estudiantes, las familias y la escuela, con el objetivo de reflexionar sobre el uso de drogas y los estilos de vida, y replantear conceptos y percepciones.

## INTRODUCTION

The Sustainable Development Goals (SDGs) are a global agenda of the United Nations, in 2015, composed of 17 goals and 169 targets to be achieved by 2030, which considers the consumption of psychoactive substances a threat to people’s well-being and quality of life, being one of the most complex problems to intervene in contemporary times. Furthermore, it commits to promoting health and achieving the SDGs, including SDG3 (ensuring healthy lives and promoting well-being for all), which establishes among its goals strengthening the prevention and treatment of psychoactive substance abuse, especially among adolescents^(1)^.

Adolescence is a period of human development marked by physical, cognitive and psychosocial transformations. In the search for challenges, independence, experimentation and affirmation of identity, adolescents may adopt behaviors and expose themselves to risks capable of affecting their physical and mental health, the learning process and social relationships^(2-4)^.

These behaviors, linked to vulnerable socioeconomic, family and cultural contexts, and weaknesses in social and health public policies, can increase adolescents’ exposure to the use of psychoactive substances. The first contact, for instance, usually occurs in early adolescence, and consumption among this population currently represents a global public health problem^(5)^.

International scientific literature points to adolescence as a critical period for the onset of psychoactive substance use, considering that the brain is still developing at this stage of life. The onset of drug use in adolescence and regular and frequent use have been associated with the risk of developing mental and behavioral disorders due to substance use, psychiatric comorbidities, and other developmental problems in adulthood. Marijuana and e-cigarettes, for instance, have become popular among adolescents in several regions of the world, and their consumption patterns have been a cause for concern^(6)^.

Brazilian adolescents live in a sociocultural context marked by daily contact with alcoholic beverages, and the understanding that their consumption during adolescence is legal represents a way of bringing people together and that there are weaknesses in the monitoring of consumption. Added to this reality is the experimentation with alcohol and other psychoactive substances, conceived as a rite of passage in a period of life permeated by intense experiences and experimentation. At this stage, new personal relationships are established and experienced through interaction in peer groups, which can generate learning and risky behaviors^(7)^.

There is evidence of the benefits of school-based interventions aimed at reducing the use of alcohol and other psychoactive substances, which makes it timely to bring health and education services closer together. In Brazil, even with consistent scientific literature on the use of psychoactive substances among adolescents, there is still a shortage of research developed or applied in school environments aimed at preventing substance abuse in adolescence and that considers the singularities of this population, such as their knowledge and perceptions on the subject^(8)^.

Knowledge about psychoactive substances is an important protective factor against consumption among adolescents, enabling the development of a critical view of reality and enabling them to take responsibility for their choices^(2)^. These individuals’ perceptions about drugs and their consumption take on different proportions and meanings in Brazil, depending on the location and cultural peculiarities. The Central-West region of Brazil, in particular, has absorbed migratory flows from all over the country of people in search of employment and better living conditions. The influence of this expansion process on adolescents’ lifestyle has been evidenced in several studies, in which this territory emerges with a large percentage of young people who have already tried alcohol and other drugs in their lives^(9)^.

In order to contribute to scientific production on SDGs, promoting adolescent health and preventing the use of psychoactive substances, the interest in carrying out this study arose from the following question: what are the perceptions of adolescents in public schools about the use of psychoactive substances?

## OBJECTIVES

To understand the perception of adolescents in public schools regarding the use of psychoactive substances.

## METHODS

### Ethical aspects

The study followed the recommendations of Resolution 466/2012 of the Brazilian National Health Council. It was approved by the *Universidade Federal de Mato Grosso do Sul* (UFMS) Research Ethics Committee, whose opinion is attached to this submission. Consent was obtained from participants, including their parents/guardians, through the signing of the Informed Consent Form and the Informed Assent Form.

### Theoretical framework

Social representations consist of discursive practices and real behaviors of social agents present in opinions, positions, manifestations or individual stances regarding a given theme/phenomenon of everyday life, and which are shared/adopted collectively in society and culture. It is possible to group and reconstruct statements or other manifestations of individual thoughts into large categories with semantically equivalent representations that portray a collective^(10)^.

Regarding the phenomenon of psychoactive substance use among adolescents, the Theory of Social Representations contributes to understanding the values, beliefs and attitudes of these individuals towards drugs. Social representations are a form of knowledge originated and shared socially that aims to construct a reality common to a given social group. As systems of interpretation that manage interpersonal relationships and people’s relationships with the world, social representations guide and organize social conduct and communication, impacting the processes that attribute meaning to everyday events^(11)^.

The findings were interpreted and discussed in light of studies that address the consumption of psychoactive substances among adolescents, especially those that had the Theory of Social Representations as a theoretical-methodological framework.

### Study design

This is an exploratory study with a qualitative approach. The study is part of a macro research project entitled “Violence at school and use of psychoactive drugs among adolescents”, linked to UFMS, which investigated the consumption of psychoactive substances and practice/victimization of/by violence (physical, psychological, verbal and sexual) among adolescents in public schools of a municipality in Mato Grosso do Sul. The COnsolidated criteria for REporting Qualitative research were used to guide the study method^(12)^.

### Study setting

The study was conducted at a state public school in a medium-sized municipality in the northern region of the state of Mato Grosso do Sul, Brazil. The municipality has a population of 32,151 inhabitants, with 14 establishments offering elementary school, five offering high school, and 11 health establishments. The school where the study was conducted offers the initial and final stages of primary education in the morning and afternoon periods.

### Data source

The study population consisted of 29 students enrolled in the 8^th^ grade of elementary school in the morning period. The school coordinators suggested that the research be conducted with this population, considering previous reports from teachers and parents/guardians of these students, specifically related to knowledge and consumption of psychoactive substances. Students aged ≥ 12 and ≤ 17 years and who were regularly enrolled in the morning period of the 8^th^ grade of elementary school were included. Those who were not present in class during the data collection period were excluded. Of the 29 students, ten refused to participate in the study and five were excluded for not attending school. Thus, 14 adolescents participated in the study.

### Data collection, organization and analysis

Data collection was carried out in November 2022, in a classroom, on a day and time provided by school administration. A self-administered sociodemographic questionnaire was used, with questions about age, sex, skin color, marital status, origin, and occupation. A focus group was also conducted, led by a moderator (a nurse with experience in coordinating groups and developing qualitative research on the use of psychoactive substances) and two observers (nursing students), who had no prior contact with participants. The focus group began with an introduction between the moderator, observers, and research participants, and an explanation of the objectives of the focus group and the rules of the group session. Then, a semi-structured script was used with the following guiding questions: what do you understand by psychoactive drugs? In what situations in your daily life do you notice the use of psychoactive drugs? What are the motivations involved in the use of psychoactive drugs among adolescents? What do you suggest for dealing with problems related to the use of psychoactive drugs? Would you like to say anything else? Finally, the summary of the session was prepared and presented to participants, and acknowledgments were introduced.

The questionnaire was administered in 5 minutes, and the focus group lasted 65 minutes. Participants’ narratives were recorded using a digital recorder. The recording content was transcribed in full and later organized according to the Discourse of the Collective Subject (DCS), a method based on the Theory of Social Representations.

Literal fragments of significant content were extracted from narratives of several different individual participants’ speeches, grouped by similarity of meanings, which represented a specific position/opinion, in addition to a grouping of key expressions to finalize the summary speeches. Key expressions consisted of literal excerpts of the speeches in their essence, cleaned of particularities, expressions and repeated ideas. Common key expressions were grouped into a central idea, representing a collective thought of participants about the phenomenon investigated^(13)^.

Chart 1 illustrates data organization according to the DCS method, specifying the 15 key expressions that emerged in the speeches and gave rise to the two central ideas of the study.

**Chart 1:** Central ideas and key expressions arising from data organization according to Discourse of the Collective Subject method, Coxim, Mato Grosso do Sul, Brazil, 2022

Central ideas	Key expressions
Knowledge about psychoactive substances and users	Psychoactive substances are dangerous and harmful
	Legal psychoactive substances are not drugs
	Lack of knowledge about the repercussions of substance use on school performance
	Lack of knowledge about legal substances as drugs
	Illegal psychoactive substances are dangerous and harmful drugs
	Naturalization of the use of legal substances and easy access to psychoactive substances among adolescents
Knowledge about psychoactive substances and users	Fear of exposing the use of psychoactive substances and use in secret
	Perception of drug users associated with individual will and criminality based on moral judgment and blame
	Segregation and punishment of users of psychoactive substances as forms of coping
Elements that contribute to the consumption of psychoactive substances in adolescence	Adolescence as a period of vulnerability for the consumption of psychoactive substances
	Curiosity, desire for new sensations, peer influence, family conflicts and participation in festive events as facilitators
	Access to and consumption of psychoactive substances in everyday life
	Knowledge about the acquisition of psychoactive substances in the territory and strategies adopted for their consumption
	Weaknesses in the monitoring of the sale and consumption of psychoactive substances to/by adolescents
	Interest in learning more about psychoactive drugs and other topics relevant to adolescent health

From the process of organizing and analyzing the data, the central ideas gave rise to two categories of meaning entitled “Knowledge about psychoactive substances and users” and “Elements that contribute to the consumption of psychoactive substances in adolescence”.

## RESULTS

Of the 14 study participants, ten were male; eight were between 14 and 15 years old; and 11 were brown. All participants were single and lived in the municipality where the school was located. In addition, 13 participants dedicated themselves exclusively to their studies, and one worked outside the home.

### Knowledge about psychoactive substances and users

The narratives suggest that participants’ perception of psychoactive substances was based on their negative effects, harm to the body and the notion of dangerousness, with rare recognition of the positive aspects of consumption. Participants mentioned some of the psychoactive substances classified as legal and illegal, highlighting their effects on the body, dangers and potential harms. In DCS 1, the question is whether legal substances, such as tobacco, are drugs, reinforcing the concept of a drug as one that causes evident psychological alterations and revealing knowledge gaps on the subject.

DCS 1 - *They are all the same, all bad, all harmful. There is marijuana, LSD* [...] *the worst is crack. The person becomes hallucinatory, crazy. There is also ecstasy* [...] *crack and heroin are worse, because they affect the mind, kill neurons* [...] *drugs can make people sleepy, laugh too much, altered, energetic, crazy, agitated. It makes people hungrier. The person becomes slower. It depends on the person’s body to make them fall asleep or not. Marijuana causes eye disease, conjunctivitis* [...] *is hookah a drug? No! The drug is marijuana. It is something that makes you crazy. I don’t know* [...]. (Participants 2, 7, 9, 14)

DCS 2 indicates gaps in knowledge about the repercussions of the use of psychoactive substances by adolescents, for instance, on school performance. This hypothesis was considered only based on the drug consumed, the most evident/immediate effects produced in the body and the knowledge of consumption by other people:

DCS 2 *- Does drug use affect school performance? Sometimes yes, sometimes no. It depends on the substance. If you get drunk, lose your mind in class, or lose your mind. If you smoke three or more drugs, you pass out here at school.* [...] *if someone finds out, yes! The person starts to change, go crazy, insane, and sleepy. It depends.* (Participants 3 and 9)

Some participants described tobacco as a drug and indicated it as a better psychoactive substance compared to others. Furthermore, as can be seen in DCS 3, factors such as access, cost, effects/sensations produced in the body and composition interfere in the acceptance of a given psychoactive substance and in the perception of whether it is a better or worse drug.

DCS 3 *- The best one is the cheapest one. There are some that are more expensive. Each drug has a different sensation. It will depend, whether it is worse or better, on the substance inside* [...] *hookah is better because it has essence. Hookah is better than all the others! It tastes like fruit, I don’t know.* (Participants 3, 4, 5, 8)

The narratives indicate that legal psychoactive substances are those with the greatest acceptance and naturalization of consumption among adolescents. Participants’ perception of these substances, their effects on the body and their ease of access contributed to their consumption. Participants’ fear of exposing their consumption of illegal psychoactive substances and their fear of being judged or punished were evident, a fact that favored their secret consumption:

DCS 4 *- Most students have already tried it. People are afraid to show themselves, to show themselves in this way. To tell their parents, to tell the police about the substances they use. They are afraid of getting beaten up and of people judging them* [...] *young people who use it, use it more in secret. Nowadays, alcoholic beverages and hookah are the most used drugs. They are easier to get. They are the ones that make you less crazy.* (Participants, 6, 12 and 13)

Participants’ perception of the psychoactive substance user was associated with individual intentionality and based on moral judgment and the blaming of this subject for such behavior. A psychoactive substance user also emerged in the narratives associated with criminality and as a subject who needs to be punished for their behavior. DCS 5 illustrates participants’ common idea about the need to segregate and punish the psychoactive substance user as a form of coping.

DCS 5 *- There is a great lack of awareness, a lack of responsibility* [...] *there are bums who smoke cigarettes at school* [...] *they smoke for the first time, they want to have fun, then they get addicted and want to sustain the habit* [...] *whoever smokes drugs should be arrested. Of course they should be arrested!* [...] *I think that people only smoke because they want to! If I call you, you go if you want! I think that people use if they want to.* (Participants 1, 3, 11, 12 and 14)

### Elements that contribute to the consumption of psychoactive substances in adolescence

Participants’ narratives indicate that adolescence is a period characterized by several factors that can favor the consumption of psychoactive substances. Elements such as curiosity, the desire to experience new sensations, peer influence, family conflicts, and participation in festive events emerged as factors that contributed to a greater vulnerability to the consumption of psychoactive substances at this stage of life. Despite this greater diversity of factors emerging in DCS 6, it was clear that the perception of the consumption of psychoactive substances as being more strongly determined by individual intentionality still predominated:

DCS 6 - *In adolescence, you become curious, wanting to discover new things, new sensations. People like to smoke, they smoke out of curiosity, to try new things, to calm down, to get high, when they get nervous.* [...] *sometimes, people smoke to forget about a problem, family problems, and then they seek refuge in the drug. They smoke to feel relaxed.* [...] *sometimes, it can start with a friend. One person who smokes can encourage others. Because of the influence of people who offer it at parties, when you start going to parties.* [...] *but it depends on the person. I have many friends who have never offered it to me. It depends on the person. If I want to smoke, I smoke. If not, it’s not because of influence. I have my own will. How does a person who smokes influence another?* (Participants 7, 10, 13 and 14)

In DCS 7, participants’ access to psychoactive substances in the territory and their daily consumption in various community spaces, including family and school environments, emerged. The singularities of psychoactive substance consumption and the strategies adopted by adolescents to ensure that consumption did not generate problems were revealed:

DCS 7 *- I’ve tried hookah, alcoholic beverages, cigarettes, those things. Not here at school. But there are bums who smoke cigarettes in the school bathroom, in the courtyard, on the court, at the back of the court. They’re more hidden places, so no one will find out. I can tell by the smell of marijuana, cigarettes. Any substance that causes addiction is prohibited at school. It’s very difficult for you to enter the school with drugs, but you can enter without any problems; here they don’t even search anything.* [...] *they use them at parties, on the street, at the leisure river, at the spa, at the amusement park, at the bar, at social events, at work, at home, at their classmates’ houses, at the construction site. Everyone here at school has a relative who uses drugs. It’s the relative who uses them, someone knows them. Sometimes, I don’t know that the relative uses them and they live with me* [...]. (Participants 1, 6, 9 and 10)

The narratives also contained descriptions of ways in which adolescents access and acquire psychoactive substances. Participants knew the routes to be taken in the territory to acquire such substances, places in the community with easier access, possibilities of purchasing via the internet and weaknesses in the monitoring of the sale and consumption of psychoactive substances to/by adolescents:

DCS 8 *- You don’t have to be an adult to buy a hookah! It depends on the store. Here in the city, if you go to the market, you can buy a lot. If you buy online, you can also buy it. I buy more at the little bar on the corner, where they sell it.* [...] *marijuana is easier to get. You go to the corner, you’ll find a “drug den”, there’s one there. If you give ten reais, you’ll get a good amount. You go to the cassava plantation, there’s a hidden* [marijuana] *plant. If you want to buy drugs, go up to the top of the neighborhood. It’s easier to get them at the gym, in some neighborhoods, on the hill. Down there, there’s a little house there.* [...] *you can get drinks more directly on the city’s main avenue, at the bar and the nightclub.* (Participants 2, 4 and 14)

Participants mentioned their interest in learning more about other factors that contribute to the use of psychoactive drugs in adolescence and also in participating in group educational activities that address other issues that permeate adolescents’ life and health. In DCS 9, participants’ interest in learning more about violence, sexuality, family conflicts and mental health emerges:

DCS 9 - *I commend you for taking the time to come and talk to us, to satisfy our curiosity, to interact. If you could do that, you could come here every month and bring us more often. So that we can learn more about drugs, it can be useful for someone as advice, and the person can ask someone for advice* [...] *other topics such as violence, individual sexuality* [...] *family problems, oppression, depression.* (Participants 3, 7 and 12).

## DISCUSSION

The findings of this study reinforce adolescence as a unique and favorable period for the consumption of psychoactive substances. Participants presented knowledge about the acquisition of psychoactive substances in the territory and occasions/spaces favorable to consumption. Fear and concern of judgment and punishment for the acquisition and consumption of psychoactive substances did not prove to be an impediment to consumption; on the contrary, they favored secret consumption, including in school environments.

Perceptions of psychoactive substances based on their effects on the body and the harm they cause have emerged, as well as perceptions of drug users associated with crime, highlighting a social imaginary permeated by stigmas and devoid of reflections on the influence of social determinants of the health-disease process, making it difficult to discern between those who need health care and those who need to be punished for illegal practices. These perceptions, partly based on lack of knowledge and/or common sense, combined with the lack of access to educational actions on the subject in school environments and adolescents’ habitual behavior of not attending health services, can reinforce their vulnerability.

Adolescents often have a tendency to praise psychoactive substances so that their consumption can be appreciated and validated by groups, increasing the likelihood of them starting to use them based on a logic of belonging^(14)^. A Brazilian study that addressed the social representations of adolescent schoolchildren enrolled in the 7^th^ and 8^th^ grades of elementary school about the consumption of alcohol and other drugs indicated that the greater the knowledge about the topic, the less permissive the attitudes towards the use of psychoactive substances and the more dissociated from stigma and prejudice the beliefs about them. These findings can contribute to health policies and practices that, through the dissemination of knowledge, promote adolescent health in school environments, prevent the consumption of alcohol and other drugs, and reduce the stigma that associates the topic with marginalization^(15)^.

Dangerousness and negative social judgment, as well as the most obvious and dangerous effects, were factors that made the use of illicit substances less accepted/naturalized by adolescents. The use of substances in secret, encouraged among peers, emerged as a factor that increased risk behavior and vulnerability.

The findings of this study support the literature regarding adolescents’ lack of recognition of legal substances as drugs, since it is common for them to report not consuming psychoactive substances, but to state that they have used/do use alcohol and tobacco products. Adolescents’ social representations of psychoactive substances are often organized around aspects related to the consequences of consumption, such as addiction, trafficking, dependence and violence. Representations of the reasons for consumption are related to the influence of other people, curiosity, family difficulties, the need for acceptance among peers and lack of information^(16,17)^.

Legal substances emerged as the most acceptable based on the perception that they cause less apparent harm, have greater social/cultural acceptance and are more accessible. Access to and use of these drugs occurred in diverse social spaces and were known to the community. In relation to illegal drugs, they occurred in spaces known to groups of adolescents. Moreover, other spaces were indicated, such as family and school environments.

As in the present investigation, a study conducted with adolescents enrolled in public schools in Paraíba, Brazil, indicates that illicit drugs are the representation of psychoactive substances. In this sense, adolescents disregard substances permitted by law as harmful, and their consumption is accepted and associated with the idea of self-control, as long as it is not excessive^(2)^.

A Brazilian study conducted with adolescents from the 7^th^ grade of elementary school to the 3^rd^ grade of high school, on social representations of alcoholic beverages and their consumption, indicated an association with drinks present in everyday life (beer) and festive events. Adolescents’ behavior in consuming alcoholic beverages was considered acceptable, being commonly associated with positive thoughts and socialization among peers^(18)^.

Another Brazilian study showed that the search for well-being was a social representation for the consumption of alcohol and tobacco by adolescents. Being well with oneself, from the perspective of these adolescents, was related to the freedom to live experiences and do what one believed to be important for oneself. The findings reveal a lack of reflection on the consequences and risks arising from this behavior, in addition to a deficit in self-care, taking into account the fact that some of these subjects had been involved in acts of violence and infraction associated with the consumption of legal substances^(19)^.

In Brazil, the criminalization of the sale of alcoholic beverages to children and adolescents, through Law 13,106 of March 17, 2015, has not been a guarantee in practice, considering that alcohol has been the psychoactive substance most frequently consumed by them^(20)^.

Another study, conducted with American adolescents, indicates that the first time a psychoactive substance was consumed was in the family, among friends or at home. The first use commonly occurred at friends’ houses, at parties, bars or at school, and together with friends^(21)^.

Adolescents consume psychoactive substances more often with friends or alone, with variations in the type of substance consumed. Most adolescents who consume alcohol, marijuana or over-the-counter medications report using them in the company of friends^(22)^.

A study conducted with Jamaican adolescents showed that the use of psychoactive substances began between the ages of 12 and 17, and that most participants had friends or family members who consumed alcohol, marijuana, and cocaine. The higher prevalence of polysubstance use was associated with the variables of beginning use of psychoactive substances in childhood and adolescence, having friends and family members with a history of drunkenness and use of illicit substances, and perception of easy access to marijuana. These data support the need for interventions that consider not only the influence of the psychoactive substance use behaviors of friends and family members, but also the perception of easy access to drugs in individuals’ social and community environments^(23)^.

Participants indicated that the period they experienced was permeated by multiple factors that may favor the consumption of psychoactive substances. The results demonstrate the ease of access and the weakness of monitoring, reinforcing the difficulty of implementing strategies and public policies to promote health and prevent the use of psychoactive substances. This scenario may increase the consumption of substances among adolescents.

A study conducted with American adolescents indicated that “feeling calm, peaceful or relaxed”, “stopping worrying about a problem or forgetting bad memories”, “helping with depression or anxiety” and “experimenting or having fun” were the most reported motivations for substance use. It also highlights that the use of psychoactive substances that begins in adolescence carries the risk of substance use disorders and fatal overdose in adulthood^(22)^.

The findings also highlight doubts, uncertainties and gaps in participants’ knowledge about psychoactive substances, their effects and their repercussions, including those related to school performance. Brazilian research on the use of psychoactive substances by adolescents and related problems in school environments revealed that repeated changes of school, grade repetition, expulsions, loss of emotional ties and poor performance in assessments are common among psychoactive substance users^(14)^.

Another study, which associated the consumption of alcohol, tobacco and other psychoactive substances in adolescents with unprotected sexual practices and school problems, pointed to a higher occurrence of absenteeism, school dropout, difficulties in teaching and learning and school violence among users of these substances^(20)^.

The narratives did not mention the determinants of the health-disease process, nor the use of psychoactive substances by adolescents as a health issue. Interest in participating in educational activities that addressed, among other topics, drug use was identified. These findings point to the need to plan actions that provide opportunities for dialogue between adolescents, family members and schools, addressing adolescents in their complexity, as being vulnerable to the structural conditions of society in various dimensions.

Research on the social representations of adolescents enrolled in elementary school regarding health and illness highlighted the significant interest of these subjects in participating in educational groups, especially in school environments, that addressed topics related to sexuality, use of psychoactive substances, the health-disease process, means of preventing health problems, sports, education and profession. The relevance of health policies and health practices in schools that promote decision-making regarding health actions and restore the citizenship of these individuals during the educational process is highlighted^(24)^.

Information obtained by adolescents about the use of psychoactive substances from sources monitored by public bodies and professional associations (schools, parents and the media) is associated with a reduction in substance use. In contrast, information obtained from informal sources (siblings, friends and the Internet) encourages the use of psychoactive substances and more intense consumption. Thus, the importance of health literacy in promoting healthy lifestyles among young people and the influence of information sources on substance use is highlighted^(25)^.

No initiatives were identified by the school or health services regarding the creation of spaces for dialogue with adolescents about the consumption of psychoactive substances in school environments, nor about any other topic of interest to participants. This scenario proves to be both conducive and challenging for the planning and development of health practices in school environments that bring together adolescents, families and the school.

Factors of various kinds, such as lack of training and information on the subject and work overload, challenge educators to implement practices to prevent the use of psychoactive substances and promote adolescent health in public schools. In addition to this, the social representations of these educators about adolescence and the use of psychoactive substances among adolescents are based on common sense notions, stigmas, prejudices and judgments, which makes it difficult to discuss the subject with adolescents and families in school environments. There are still few systematized strategies by educators to prevent the use of psychoactive substances in schools, and most of those that do exist do not address the topic in a broad health context^(26)^.

Education and health actions focused on school environments, based on the purpose of a sustainable society, could consider local and regional contexts, in order to create favorable conditions for students, family members, educators and school administrators to critically reflect on problems that affect adolescents’ humanity and lives, redefining socio-environmental concepts and attitudes and rethinking lifestyles. Through participatory, horizontal and intersectoral strategies, it is possible to strengthen the commitment and implementation of sustainable behaviors, in addition to creating a culture of environmental responsibility in schools and in the community. New global demands require global actions, from a perspective of local resolution, in line with global agendas, such as the SDGs^(27)^.

The use of psychoactive substances is a global problem that is interconnected with various aspects of sustainable development. Its analysis and subsequent response, through the SDGs, demonstrate this interaction, encompassing the nature and dynamics of the problem at both the individual and collective levels. Programs focused on prevention, treatment, care, rehabilitation and social reintegration play an essential role, and programs based on scientific evidence can reduce the negative repercussions of drug use^(28)^.

Strategies for health promotion and prevention of psychoactive substance use to be planned and developed in school environments can promote spaces for dialogue on early onset of consumption, family encouragement of use, advertising and marketing of substances, availability and access. It is imperative that such strategies strengthen intersectoral collaboration in order to protect adolescents from psychoactive substance use and related harms^(29)^.

It is also worth highlighting the strategic role of Primary Health Care in strengthening public policies and the use of care technologies in humanized and comprehensive health care for adolescents and their families, based on the recognition of these as subjects of rights. At a collective level, health promotion strategies emerge as an opportunity to articulate educational practices in health, with a view to healthy choices and the development of autonomy and self-care with regard to the prevention and consumption of psychoactive substances^(20)^.

### Study limitations

The limitations of this study include: only one focus group session, which required further investigation of the information in other meetings; the study was conducted in a school environment, which could interfere with participants’ responses; and only adolescents enrolled in the 8^th^ grade of elementary school in the morning. The use of a focus group as a data collection technique may have inhibited some participants from expressing their opinions, especially in cases where they disagreed with the ideas of other group members.

### Contributions to health and nursing

The study expands and deepens the scientific literature on adolescents’ perceptions of the use of psychoactive substances, highlighting the need to enhance school environments as a space for the development of actions to promote mental health, prevent substance use and health education. The use of psychoactive substances is a barrier to achieving the SDGs. From this perspective, health and nursing professionals stand out with unparalleled responsibility and commitment in the process of social transformation, participating in the planning and implementation of policies and practices to promote health and prevent the use of psychoactive substances, with an interdisciplinary, interprofessional and intersectoral nature, which target the various actors that permeate school environments.

## FINAL CONSIDERATIONS

Adolescents’ perceptions about the use of psychoactive substances revealed judgments, doubts and knowledge gaps. Participants’ daily lives were permeated by the greater acceptance of legal chemical substances, easy access and consumption in the territory, including in school environments. It is necessary to enhance school environments with participatory, horizontal and intersectoral actions to promote health, health education and prevention of the use of psychoactive substances that include students, family members and school, aiming at reflection on drug use and lifestyles, and the redefinition of concepts and attitudes.

## Data Availability

*https://doi.org/10.48331/scielodata.LRC0JH*
