# Supplementary material for: Adolescents’ discourse on the consumption of psychoactive substances in schools
Source: Rev Bras Enferm. 2025 Nov 7;78(4):e20240269. doi: 10.1590/0034-7167-2024-0269 (PMC12594480; doi:10.1590/0034-7167-2024-0269)
Supplement: Translation

# Translation: Discurso de adolescentes sobre o consumo de substâncias psicoativas no contexto escolar

**DOI:** 10.1590/0034-7167-2024-0269pt

**Authors:** Karla Nataly Ferreira Vasconcelos, Helder de Pádua Lima, Nathan Aratani, Bianca Cristina Ciccone Giacon-Arruda, Soraia Geraldo Rozza, Sarah Lima Verde da Silva, Eysler Gonçalves Maia Brasil, Flavia Milene de Souza Nobre

**Affiliations:** I Fundação Universidade Federal de Mato Grosso do Sul. Coxim, Mato Grosso do Sul, Brasil; II Secretaria da Saúde do Ceará. Fortaleza, Ceará, Brasil; III Universidade da Integração Nacional da Lusofonia Afro-Brasileira. Redenção, Ceará, Brasil; IV Faculdade Estácio de Alagoinhas. Alagoinhas, Bahia, Brasil

**Keywords:** Promoção da Saúde, Saúde Mental, Percepção, Adolescente, Psicotrópicos.

## Abstract

**Objetivos:**

conhecer a percepção de adolescentes inseridos no contexto escolar público sobre o uso de substâncias psicoativas.

**Métodos:**

estudo exploratório qualitativo, realizado em uma escola pública com 14 adolescentes. Os dados foram coletados através de questionário sociodemográfico autoaplicável e grupo focal, no qual se utilizou um roteiro semiestruturado de perguntas, organizados conforme o Discurso do Sujeito Coletivo.

**Resultados:**

as percepções dos adolescentes sobre o uso de substâncias psicoativas revelaram julgamentos, dúvidas e lacunas de conhecimento. Evidenciou-se o cotidiano dos participantes permeado pela maior aceitação, fácil acesso e consumo de substâncias químicas lícitas no território, inclusive no ambiente escolar.

**Considerações Finais:**

faz-se necessário potencializar o ambiente escolar com ações participativas, horizontais e intersetoriais de prevenção e promoção da saúde que incluam estudantes, familiares e escola, visando à reflexão sobre uso de drogas e estilos de vida, e à ressignificação de conceitos e percepções.

## INTRODUÇÃO

Os Objetivos de Desenvolvimento Sustentável (ODS) são uma agenda global das Nações Unidas, em 2015, composta por 17 objetivos e 169 metas a serem atingidos até 2030, que considera o consumo de substâncias psicoativas uma ameaça ao bem-estar e à qualidade de vida das pessoas, sendo um dos problemas de maior complexidade de intervenção na contemporaneidade. Além disso, compromete à promoção da saúde e o alcance dos ODS, incluindo o ODS3 (assegurar uma vida saudável e promover o bem-estar de todos), o qual estabelece entre suas metas reforçar a prevenção e o tratamento do abuso de substâncias psicoativas, em especial entre adolescentes^(1)^.

A adolescência é um período do desenvolvimento humano marcado por transformações físicas, cognitivas e psicossociais. Na busca por desafios, independência, experimentações e afirmação da identidade, o adolescente pode adotar comportamentos e se expor a riscos capazes de repercutir na saúde física e mental, no processo de aprendizagem e nas relações sociais^(2-4)^.

Esses comportamentos, atrelados aos contextos socioeconômicos, familiares e culturais vulneráveis e a fragilidades de políticas públicas sociais e de saúde, podem potencializar a exposição do adolescente ao consumo de substâncias psicoativas. O primeiro contato, por exemplo, ocorre geralmente no início da adolescência, e o consumo entre essa população representa um problema de saúde pública mundial na atualidade^(5)^.

A literatura científica internacional aponta a adolescência como um período crítico para o início do consumo de substâncias psicoativas, considerando que o cérebro ainda se encontra em desenvolvimento nessa etapa da vida. O início do consumo de drogas na adolescência e o uso regular e frequente têm sido associados ao risco de desenvolvimento de transtornos mentais e comportamentais devido ao uso de substâncias, comorbidades psiquiátricas e outros problemas de desenvolvimento na idade adulta. Maconha e cigarros eletrônicos, por exemplo, têm se popularizado entre adolescentes em várias regiões do mundo, e seu padrão de consumo tem sido motivo de preocupação^(6)^.

Adolescentes brasileiros estão inseridos em um contexto sociocultural marcado pela convivência cotidiana com bebidas alcoólicas, e o entendimento de que seu consumo na adolescência é lícito representa uma forma de agregar pessoas e de que há fragilidades na fiscalização do consumo. Soma-se a essa realidade a experimentação de álcool e outras substâncias psicoativas, concebida como um rito de passagem em um período da vida permeado por intensas experiências e experimentações. Nessa fase, novas relações pessoais são estabelecidas e vivenciadas através da interação em grupos de pares, podendo gerar aprendizados e comportamentos de risco^(7)^.

Há evidências dos benefícios de intervenções no âmbito escolar direcionadas para a redução do uso de álcool e outras substâncias psicoativas, o que torna oportuna a aproximação entre serviços de saúde e educação. No Brasil, mesmo com a consistente literatura científica que versa sobre o consumo de substâncias psicoativas entre adolescentes, ainda há uma escassez de pesquisas desenvolvidas ou aplicadas no ambiente escolar voltadas para a prevenção do abuso de substâncias na adolescência e que considerem as singularidades desse público, como seus conhecimentos e percepções sobre o tema^(8)^.

Conhecer sobre o tema substâncias psicoativas constitui em um importante fator de proteção para o consumo entre adolescentes, possibilitando o desenvolvimento de uma visão crítica da realidade e capacitando-os a assumir responsabilidades por suas escolhas^(2)^. As percepções desses sujeitos sobre drogas e seu consumo ganham proporções e significados diferentes no território brasileiro, dependendo da localidade e das peculiaridades culturais. A região Centro-Oeste do Brasil, em particular, tem absorvido fluxos migratórios de todo o país de pessoas em busca de emprego e melhores condições de vida. A influência desse processo de expansão no estilo de vida de adolescentes tem sido evidenciada em várias pesquisas, nas quais esse território emerge com grande percentual de jovens que já experimentaram álcool e outras drogas na vida^(9)^.

De modo a contribuir para a produção científica sobre ODS, promoção da saúde de adolescentes e prevenção do uso de substâncias psicoativas, surgiu o interesse em realizar este estudo a partir do seguinte questionamento: quais as percepções de adolescentes inseridos no contexto escolar público sobre o uso de substâncias psicoativas?

## OBJETIVOS

Conhecer a percepção de adolescentes inseridos no contexto escolar público sobre o uso de substâncias psicoativas.

## MÉTODOS

### Aspectos éticos

O estudo seguiu as recomendações da Resolução n^o^ 466/2012 do Conselho Nacional de Saúde. Foi aprovado pelo Comitê de Ética em Pesquisa da Universidade Federal de Mato Grosso do Sul (UFMS), cujo parecer foi anexado à presente submissão. O consentimento foi obtido dos participantes, incluindo seus pais/responsáveis, por meio da assinatura do Termo de Consentimento Livre e Esclarecido e Termo de Assentimento Livre e Esclarecido.

### Referencial teórico

As representações sociais consistem em práticas discursivas e comportamentos reais de agentes sociais presentes em opiniões, posicionamentos, manifestações ou posicionamentos individuais a respeito de um dado tema/fenômeno da vida cotidiana, e que são compartilhados/adotados coletivamente na sociedade e na cultura. É possível agrupar e reconstituir depoimentos ou outras manifestações de pensamentos individuais em grandes categorias com representações semanticamente equivalentes e que retratam uma coletividade^(10)^.

No tocante ao fenômeno do consumo de substâncias psicoativas entre adolescentes, a Teoria das Representações Sociais contribui na compreensão de valores, crenças e atitudes desses sujeitos diante do objeto drogas. As representações sociais são uma forma de conhecimento originada e compartilhada socialmente que objetiva a construção de uma realidade comum a um determinado grupo social. Como sistemas de interpretação que gerenciam as relações interpessoais e das pessoas com o mundo, as representações sociais guiam e organizam condutas e comunicações sociais, repercutindo nos processos que atribuem sentidos aos eventos cotidianos^(11)^.

Os achados foram interpretados e discutidos à luz de estudos que abordam o consumo de substâncias psicoativas entre adolescentes, sobretudo aqueles que tiveram a Teoria das Representações Sociais como fundamentação teórico-metodológica.

### Tipo de estudo

Trata-se de estudo de caráter exploratório com abordagem qualitativa. O estudo integra um macroprojeto de pesquisa intitulado “Violência na escola e uso de drogas psicoativas entre adolescentes”, vinculado à UFMS, que investigou o consumo de substâncias psicoativas e a prática/vitimização de/por violências (física, psicológica, verbal e sexual) em adolescentes no contexto escolar público de um município sul-mato-grossense. Para nortear o método do estudo, foi utilizado o *COnsolidated criteria for REporting Qualitative research* ^(12)^.

### Cenário do estudo

O estudo foi realizado em uma escola pública estadual de um município de médio porte da região Norte do estado de Mato Grosso do Sul, Brasil. O município possui uma população de 32.151 habitantes, contando com 14 estabelecimentos que ofertam o ensino fundamental, cinco que oferecem o ensino médio, além de 11 estabelecimentos de saúde. A escola na qual o estudo foi conduzido oferta as etapas inicial e final do ensino fundamental nos períodos matutino e vespertino.

### Fonte de dados

A população do estudo foi representada pelos 29 estudantes matriculados no 8º ano do ensino fundamental, período matutino. A coordenação da escola sugeriu que a pesquisa fosse realizada com essa população, considerando os relatos prévios de professores e pais/responsáveis desses estudantes, em específico, relacionados ao conhecimento e consumo de substâncias psicoativas. Foram incluídos estudantes com idade ≥ 12 e ≤ 17 anos e que estavam regularmente matriculados no período matutino do 8º ano do ensino fundamental. Excluíram-se aqueles que não estiveram presentes em sala de aula no período de coleta de dados. Dos 29 estudantes, dez se recusaram a participar do estudo e cinco foram excluídos por não comparecem à escola. Dessa maneira, participaram do estudo 14 adolescentes.

### Coleta, organização e análise dos dados

A coleta de dados foi realizada em novembro de 2022, em sala de aula, com dia e horário disponibilizados pela direção da escola. Utilizou-se um questionário sociodemográfico autoaplicável com questionamentos sobre idade, sexo, cor da pele, estado civil, procedência e ocupação. Também foi realizado grupo focal, conduzido por um moderador (enfermeiro com experiência em coordenação de grupos e desenvolvimento de pesquisas qualitativas sobre uso de substâncias psicoativas) e dois observadores (estudantes de enfermagem), que não tiveram contato prévio com os participantes. Iniciou-se o grupo focal com a apresentação entre moderador, observadores e participantes da pesquisa, e a exposição dos objetivos do grupo focal e das regras da sessão grupal. Em seguida, utilizou-se um roteiro semiestruturado com as seguintes questões norteadoras: o que você entende por drogas psicoativas? Em quais situações do seu cotidiano você percebe o uso de drogas psicoativas? Quais as motivações envolvidas no uso de drogas psicoativas entre adolescentes? O que você sugere para se lidar com problemas relacionados ao consumo de drogas psicoativas? Desejam falar algo mais? Por fim, foi elaborada e apresentada aos participantes a síntese da sessão, sendo feitos os agradecimentos.

A aplicação do questionário ocorreu em 5 minutos, e o grupo focal teve duração de 65 minutos. As narrativas dos participantes foram registradas por um gravador digital. O conteúdo da gravação foi transcrito na íntegra e, posteriormente, organizado segundo o Discurso do Sujeito Coletivo (DSC), método fundamentado na Teoria das Representações Sociais.

Foram extraídos fragmentos literais de conteúdo significativo das narrativas de vários e diferentes discursos individuais dos participantes, reunidos por similaridade de sentidos, que representavam um posicionamento/opinião específico, além de um agrupamento de expressões-chave para finalizar os discursos-síntese. As expressões-chave consistiam em trechos literais das falas em sua essência, limpos de particularidades, expressões e ideias repetidas. Expressões-chave comuns foram agrupadas em uma ideia central, representando um pensamento coletivo dos participantes sobre o fenômeno investigado^(13)^.

O Quadro 1 ilustra a organização dos dados segundo o método DSC, especificando as 15 expressões-chave que emergiram nos discursos e originaram as duas ideias centrais do estudo.

**Quadro 1:** Ideias centrais e expressões-chave oriundas da organização dos dados segundo o método Discurso do Sujeito Coletivo, Coxim, Mato Grosso do Sul, Brasil, 2022

Ideias centrais	Expressões-chave
Conhecimentos sobre substâncias psicoativas e usuários	Substâncias psicoativas são perigosas e danosas
	Substâncias psicoativas lícitas não são drogas
	Desconhecimento sobre as repercussões do consumo de substâncias no desempenho escolar
	Desconhecimento sobre substâncias lícitas como drogas
	Substâncias psicoativas ilícitas são drogas perigosas e danosas
Conhecimentos sobre substâncias psicoativas e usuários	Naturalização do uso de substâncias lícitas e facilidade de acesso a substâncias psicoativas entre adolescentes
	Temor em expor o consumo de substâncias psicoativas e consumo às escondidas
	Percepção do usuário de drogas associada à vontade individual e criminalidade fundamentada no julgamento moral e na culpabilização
	Segregação e punição do usuário de substâncias psicoativas como formas de enfrentamento
Elementos que concorrem para o consumo de substâncias psicoativas na adolescência	Adolescência como período de vulnerabilidade para o consumo de substâncias psicoativas
	Curiosidade, desejo de novas sensações, influência dos pares, conflitos familiares e participação em eventos festivos como facilitadores
	Acesso e consumo de substâncias psicoativas no cotidiano
	Conhecimento sobre aquisição de substâncias psicoativas no território e estratégias adotadas para o consumo
	Fragilidades na fiscalização da venda e do consumo de substâncias psicoativas para/por adolescentes
	Interesse em conhecer mais sobre drogas psicoativas e outros temas pertinentes à saúde do adolescente

A partir do processo de organização e análise dos dados, as ideias centrais originaram duas categorias de sentido intituladas “Conhecimentos sobre substâncias psicoativas e usuários” e “Elementos que concorrem para o consumo de substâncias psicoativas na adolescência”.

## RESULTADOS

Dos 14 participantes do estudo, dez eram do sexo masculino; oito tinham idade entre 14 e 15 anos; e 11 tinham cor parda. Todos os participantes eram solteiros e residiam no município no qual a escola estava localizada. Além disso, 13 participantes se dedicavam exclusivamente aos estudos, e um realizava trabalho externo ao domicílio.

### Conhecimentos sobre substâncias psicoativas e usuários

As narrativas sugerem que a percepção dos participantes acerca das substâncias psicoativas se fundamentava nos efeitos negativos, danos ocasionados ao organismo e na noção de periculosidade, com raro reconhecimento de aspectos positivos do consumo. Os participantes mencionaram algumas das substâncias psicoativas classificadas como lícitas e ilícitas, destacando seus efeitos no organismo, perigos e potenciais danos. No DSC 1, questiona-se se substâncias lícitas, como o tabaco, são drogas, reforçando o conceito de droga como sendo aquela que causa efeitos de alterações psíquicas evidentes e revelando lacunas de conhecimento sobre o tema.

DSC 1 - *São todas iguais, todas ruins, todas fazem mal. É maconha, LSD* [...] *a pior é o crack. A pessoa fica alucinada, doida. Também tem o ecstasy* [...] *crack e heroína são piores, pois afetam a mente, matam os neurônios* [...] *as drogas podem deixar as pessoas com sonolência, rindo demais, alteradas, energéticas, doidas, agitadas. Dá mais fome. A pessoa fica mais lenta. Depende do organismo da pessoa para fazer adormecer ou não. Maconha dá doença no olho, conjuntivite* [...] *narguilé é droga? Não! Droga é maconha. É uma coisa que deixa doido. Eu não sei* [...]. (Participantes 2, 7, 9, 14)

O DSC 2 denota lacunas de conhecimento sobre as repercussões do uso de substâncias psicoativas por adolescentes, por exemplo, no desempenho escolar. Essa hipótese foi considerada apenas com base na droga consumida, nos efeitos mais evidentes/imediatos produzidos no organismo e na ciência do consumo por parte de outras pessoas:

DSC 2 *- Se o uso de droga afeta o desempenho escolar? Às vezes, sim, às vezes, não. Depende da substância. Se vai ficar bêbado, “despirocar” dentro da sala, ficar fora de si. Se fumar uns três, desmaiar aqui na escola.* [...] *se alguém descobrir, sim! A pessoa começa alterar, ficar doida, louca, ficar com sono. Vai depender.* (Participantes 3 e 9)

Alguns participantes descreveram o tabaco como uma droga, e o indicaram como uma substância psicoativa melhor em comparação a outras. Ademais, como é possível observar no DSC 3, fatores como acesso, valor de custo, efeitos/sensações produzidos no organismo e composição interferem na aceitação de determinada substância psicoativa e na percepção de melhor ou pior droga.

DSC 3 *- A melhor é a mais barata. Tem umas que são mais caras. Cada droga tem uma sensação diferente. Vai depender, se pior ou melhor, da substância que tem dentro* [...] *o narguilé é melhor porque tem essência. Narguilé é melhor do que todas as outras! Tem gosto de frutas, sei lá.* (Participantes 3, 4, 5, 8)

As narrativas indicam as substâncias psicoativas lícitas como aquelas com maior aceitação e naturalização do consumo entre adolescentes. A percepção dos participantes sobre essas substâncias, os efeitos produzidos no organismo e a facilidade de acesso contribuíam para o consumo delas. Evidenciaram-se o receio dos participantes em expor o consumo de substâncias psicoativas ilícitas e o medo de sofrer julgamentos ou punições, fato que favorecia o consumo às escondidas:

DSC 4 *- A maioria dos estudantes já experimentou. O povo tem medo de mostrar, de se mostrar assim. De contar para o pai, para a polícia as substâncias que usa. Medo de levar uma coça e das pessoas julgarem* [...] *os jovens que usam, usam mais escondidos. Bebidas alcoólicas e narguilé, hoje em dia, estão sendo as drogas mais usadas. São mais fáceis de conseguir. São as que deixam menos doido.* (Participantes, 6, 12 e 13)

A percepção dos participantes sobre o usuário de substâncias psicoativas se mostrou associada à intencionalidade individual e fundamentada no julgamento moral e na culpabilização desse sujeito por tal comportamento. O usuário de substâncias psicoativas também emergiu nas narrativas associado à criminalidade e como sujeito que precisa ser punido por seu comportamento. O DSC 5 ilustra a ideia comum dos participantes sobre a necessidade de segregar e punir o usuário de substâncias psicoativas como forma de enfrentamento.

DSC 5 *- É muita falta de consciência, é falta de responsabilidade* [...] *tem vagabundo que fuma cigarro na escola* [...] *ele fuma a primeira vez, vai se divertir, aí vicia e quer sustentar o vício* [...] *quem fuma droga deve ser preso. Lógico que tem que ser preso!* [...] *eu acho que a pessoa só fuma por vontade própria! Se eu te chamar, você vai se quiser! Eu acho que a pessoa usa se quiser.* (Participantes 1, 3, 11, 12 e 14)

### Elementos que concorrem para o consumo de substâncias psicoativas na adolescência

As narrativas dos participantes denotam a adolescência como um período caracterizado por múltiplos fatores que podem favorecer o consumo de substâncias psicoativas. Elementos como a curiosidade, o desejo de experimentar novas sensações, a influência entre pares, os conflitos familiares e a participação em eventos festivos emergiram como fatores que concorriam para uma maior vulnerabilidade ao consumo de substâncias psicoativas nessa fase da vida. Apesar dessa maior diversidade de fatores emergir no DSC 6, foi notório que ainda predominava a percepção do consumo de substâncias psicoativas determinado mais fortemente à intencionalidade individual:

DSC 6 - *Na adolescência, você fica curiosa, querendo descobrir coisas novas, sensações novas. A pessoa gosta de usar, usa por curiosidade, para tentar experimentar coisas novas, se acalmar, para ficar doidão, quando fica nervosa.* [...] *às vezes, a pessoa fuma para esquecer algum problema, problemas familiares, aí busca um refúgio na droga. Ele usa para se sentir relaxado.* [...] *às vezes, pode começar por um colega. Uma pessoa que fuma pode incentivar várias. Por influência das pessoas que ficam oferecendo em festas, quando você começa a ir para festas.* [...] *mas depende da pessoa. Eu tenho muitos amigos que nunca me ofereceram. Depende da pessoa. Se eu quiser fumar, eu fumo. Se não, não é por causa de influência. Eu tenho vontade própria. Como que a pessoa fumando influencia outra?* (Participantes 7, 10, 13 e 14)

No DSC 7, emergiram o acesso dos participantes a substâncias psicoativas no território e o consumo cotidiano em diversos espaços comunitários, incluindo nos ambientes familiar e escolar. Revelam-se as singularidades do consumo de substâncias psicoativas e as estratégias adotadas por adolescentes para que o consumo não gerasse problemas:

DSC 7 *- Eu já experimentei narguilé, bebidas alcoólicas, cigarro, essas coisas. Aqui na escola, não. Mas tem vagabundo que fuma cigarro ali no banheiro da escola, no pátio, na quadra, no fundo da quadra. São lugares mais escondidos, para ninguém descobrir. Eu sei pelo cheiro de maconha, de cigarro. Qualquer substância que causa vício é proibida na escola. É muito difícil você entrar dentro da escola com droga, mas você pode entrar de boa; aqui nem revistam nada.* [...] *usam em festa, na rua, no rio de lazer, no balneário, no parque de diversão, no bar, em sociais, no trabalho, em casa mesmo, na casa dos colegas, na construção. Todo mundo aqui na escola tem algum parente que usa droga. É o parente que usa, alguém conhece sim. Às vezes, não sei que o parente usa e ele mora comigo* [...]. (Participantes 1, 6, 9 e 10)

As narrativas também continham a descrição de formas de acesso e aquisição de substâncias psicoativas por parte de adolescentes. Os participantes conheciam caminhos a serem percorridos no território para adquirir tais substâncias, locais na comunidade com maior facilidade de acesso, possibilidades de compra através da *internet* e fragilidades na fiscalização da venda e do consumo de substâncias psicoativas para/por adolescentes:

DSC 8 *- Não precisa ser maior de idade para comprar narguilé! Depende da loja. Aqui na cidade, se você for na feira, você compra um monte. Se comprar pela internet, também vende. Eu compro mais no barzinho da esquina, lá vende mesmo.* [...] *a maconha é mais fácil de conseguir. Você vai ali na esquina, você encontra uma “biqueira”, tem lá. Se você der dez reais, leva uma boa quantidade. Você vai na plantação de mandioca, tem um pé* [de maconha] *escondido. Você quer comprar droga, vai lá para cima do bairro. Mais fácil é conseguir no ginásio, em alguns bairros, no morro. Lá na descida, tem uma casinha lá.* [...] *a bebida é mais direto na avenida principal da cidade, no bar e na boate.* (Participantes 2, 4 e 14)

Os participantes mencionaram o interesse em conhecer mais sobre outros elementos que concorrem para o uso de drogas psicoativas na adolescência e, também, em participar de ações educativas grupais que abordassem outros temas que permeiam a vida e a saúde do adolescente. No DSC 9, emerge o interesse dos participantes em conhecer mais sobre violências, sexualidade, conflitos familiares e saúde mental:

DSC 9 - *Elogio vocês por terem tirado o tempo de vocês para vir conversar com nós, tirar nossa curiosidade, interagir. Se pudesse ter isso, ficar vindo para cá a cada mês e trazer mais vezes. Para a gente saber mais sobre as drogas, pode servir para alguém como conselho, e a pessoa pode pedir para alguém um conselho* [...] *outros temas como violência, sexualidade do indivíduo* [...] *problemas familiares, opressão, depressão.* (Participantes 3, 7 e 12).

## DISCUSSÃO

Os achados do presente estudo reforçam a adolescência como um período singular e propício para o consumo de substâncias psicoativas. Os participantes apresentaram conhecimento sobre aquisição de substâncias psicoativas no território e ocasiões/espaços favoráveis ao consumo. O medo e o receio de julgamentos e punições pela aquisição e consumo de substâncias psicoativas não se mostraram impeditivos para o consumo; pelo contrário, favoreciam o consumo às escondidas, inclusive no ambiente escolar.

Emergiram percepções de substâncias psicoativas baseadas nos efeitos produzidos no organismo e danos ocasionados, além de percepções sobre usuários de drogas associadas à criminalidade, evidenciando um imaginário social permeado por estigmas e desprovido de reflexões sobre a influência dos determinantes sociais do processo de saúde-doença, dificultando o discernimento entre quem necessita de assistência em saúde e quem precisa ser punido por práticas ilegais. Essas percepções, em parte fundamentadas no desconhecimento e/ou senso comum, aliadas à falta de acesso a ações educativas sobre o tema no ambiente escolar e ao comportamento habitual de adolescentes de não comparecer a serviços de saúde, podem reforçar sua vulnerabilidade.

Frequentemente, adolescentes têm uma tendência a enaltecer as substâncias psicoativas, de forma que seu consumo pode ser apreciado e validado por grupos, aumentando a probabilidade de iniciarem o uso a partir de uma lógica de pertencimento^(14)^. Estudo brasileiro que abordou as representações sociais de adolescentes escolares matriculados no 7º e 8º ano do ensino fundamental sobre o consumo de álcool e outras drogas apontou que, quanto maior o conhecimento acerca do tema, menos permissivas são as atitudes em relação ao uso de substâncias psicoativas e mais dissociadas de estigmas e preconceitos as crenças a respeito delas. Esses achados podem contribuir para políticas e práticas de saúde que, através da difusão do conhecimento, promovam a saúde do adolescente no ambiente escolar, previnam o consumo de álcool e outras drogas, e reduzam o estigma que associa a temática à marginalização^(15)^.

A periculosidade e o julgamento social negativo, bem como os efeitos mais evidentes e considerados perigosos, foram fatores que tornaram o uso de substâncias ilícitas menos aceito/naturalizado pelos adolescentes. O consumo de substâncias às escondidas, incentivado entre pares, surgiu como fator potencializador de comportamentos de risco e vulnerabilidade.

Os achados do presente estudo corroboram a literatura no tocante à falta de reconhecimento dos adolescentes sobre as substâncias lícitas como drogas, visto que é comum esses relatarem não consumir substâncias psicoativas, mas afirmarem ter feito/fazer uso de álcool e derivados do tabaco. As representações sociais de adolescentes sobre substâncias psicoativas frequentemente se organizam em torno de aspectos relativos às consequências do consumo como, como vício, tráfico, dependência e violência. As representações para os motivos do consumo mantêm relação com a influência de outras pessoas, curiosidade, dificuldades familiares, necessidade de aceitação entre pares e falta de informação^(16,17)^.

Substâncias lícitas emergiram como as mais aceitáveis com base na percepção de que causam menos danos aparentes, têm maior aceitação social/cultural e são mais acessíveis. O acesso e o uso dessas drogas ocorriam em espaços sociais diversos e de conhecimento da comunidade. Em relação às ilícitas, ocorriam em espaços de conhecimento de grupos de adolescentes. Ademais, outros espaços foram indicados, como os ambientes familiar e escolar.

Assim como na presente investigação, estudo realizado com adolescentes matriculados em escolas públicas da Paraíba, Brasil, indica que as drogas ilícitas são a representação das substâncias psicoativas. Nesse sentido, adolescentes desconsideram substâncias permitidas por lei como danosas, e seu consumo é aceito e associado à ideia de autocontrole, desde que não ocorra em excesso^(2)^.

Estudo brasileiro desenvolvido com adolescentes do 7º ano do ensino fundamental ao 3º ano do ensino médio, sobre representações sociais de bebidas alcoólicas e seu consumo, indicou associação a bebidas presentes no cotidiano (cerveja) e eventos festivos. O comportamento do adolescente em consumir bebidas alcoólicas foi considerado aceitável, sendo comumente associado a pensamentos positivos e socialização entre pares^(18)^.

Outra investigação brasileira evidenciou a busca por bem-estar como representação social para o consumo de álcool e tabaco por adolescentes. Estar bem consigo mesmo, na perspectiva desses adolescentes, relacionava-se à liberdade de viver experiências e fazer o que se acreditava ser importante para si. Os achados revelam falta de reflexão sobre as consequências e riscos decorrentes desse comportamento, além de déficit no autocuidado, levando em consideração o fato de alguns desses sujeitos terem se envolvido em atos de violência e de infração associado ao consumo de substâncias lícitas^(19)^.

No Brasil, a criminalização da venda de bebida alcoólica para crianças e adolescentes, por meio da Lei n^o^ 13.106, de 17 de março de 2015, não tem sido uma garantia na prática, considerando que o álcool tem sido a substância psicoativa mais frequentemente consumida por eles^(20)^.

Outra investigação, conduzida com adolescentes norte-americanos, aponta que a primeira obtenção de uma substância psicoativa de consumo se deu na família, entre amigos ou no próprio domicílio. O primeiro uso ocorreu comumente na casa de amigos, festas, bares ou na escola, e juntamente com amigos^(21)^.

Adolescentes consomem substâncias psicoativas, mais frequentemente, acompanhados de amigos ou sozinhos, havendo variações para o tipo de substância consumida. A maioria dos adolescentes que consume álcool, maconha ou medicamentos sem prescrição médica relata o uso na companhia de amigos^(22)^.

Pesquisa realizada com adolescentes jamaicanos evidenciou o início do uso de substâncias psicoativas entre 12 e 17 anos de idade, sendo que a maioria dos participantes tinha amigos ou familiares que consumiam bebidas alcoólicas, maconha e cocaína. A maior prevalência do uso de múltiplas substâncias esteve associada às variáveis início do uso de substâncias psicoativas na infância e adolescência, ter amigos e familiares com histórico de embriaguez e uso de substâncias ilícitas, e percepção da facilidade no acesso à maconha. Esses dados endossam a necessidade de intervenções que considerem não apenas a influência dos comportamentos de uso de substâncias psicoativas de amigos e familiares, mas, também, a percepção da facilidade de acesso às drogas no ambiente social e comunitário do indivíduo^(23)^.

Foi indicado pelos participantes que o período por eles vivenciado é permeado por múltiplos fatores que podem favorecer o consumo de substâncias psicoativas. Os resultados demonstram a facilidade do acesso e fragilidade de fiscalização, reforçando a dificuldade de implementação de estratégias e políticas públicas de promoção da saúde e prevenção do uso de substâncias psicoativas. Esse cenário pode potencializar o consumo de substâncias entre os adolescentes.

Estudo desenvolvido com adolescentes norte-americanos apontou o “sentir-se tranquilo, calmo ou relaxado”, “parar de se preocupar com um problema ou esquecer lembranças ruins”, “ajudar na depressão ou na ansiedade” e “experimentar ou se divertir” como as motivações mais relatadas para o consumo de substâncias. Ressalta, ainda, que o consumo de substâncias psicoativas iniciado na adolescência traz risco de transtornos relacionados ao uso de substâncias e de overdose fatal na idade adulta^(22)^.

Os achados também evidenciam dúvidas, incertezas e lacunas de conhecimento dos participantes sobre substâncias psicoativas, efeitos produzidos pelas mesmas e suas repercussões, incluindo aquelas relacionadas ao desempenho escolar. Pesquisa brasileira acerca do consumo de substâncias psicoativas por adolescentes e dos problemas relacionados no ambiente escolar revelou que são comuns as mudanças repetidas de escola, repetências, expulsões, perda de vínculos afetivos e baixo desempenho em avaliações entre usuários de substâncias psicoativas^(14)^.

Outro estudo, que associou o consumo de álcool, tabaco e outras substâncias psicoativas em adolescentes com práticas sexuais desprotegidas e problemas escolares, apontou uma maior ocorrência de absenteísmo, evasão escolar, dificuldades no ensino-aprendizagem e violência escolar entre os usuários dessas substâncias^(20)^.

Nas narrativas, não houve menção aos determinantes do processo de saúde-doença, nem ao consumo de substâncias psicoativas por adolescentes como questão de saúde. Identificou-se o interesse em participar de ações educativas que abordassem, entre outros temas, o consumo de drogas. Esses achados apontam para a necessidade de planejamento de ações que oportunizem o diálogo entre adolescentes, familiares e escolas, abordando o adolescente em sua complexidade, como ser vulnerabilizado pelas condições estruturais da sociedade em diversas dimensões.

Pesquisa sobre as representações sociais de adolescentes matriculados no ensino fundamental sobre saúde e doença destacou o significativo interesse desses sujeitos em participar de grupos educativos, sobretudo no ambiente escolar, que abordassem temas relativos à sexualidade, uso de substâncias psicoativas, processo de saúde-doença, meios de prevenção de agravos à saúde, esporte, educação e profissão. Ressalta-se a relevância de políticas de saúde e práticas de saúde nas escolas que promovam a tomada de decisão em relação às ações de saúde e resgatem a cidadania desses indivíduos durante o processo educativo^(24)^.

Tem-se que a informação obtida por adolescentes sobre o consumo de substâncias psicoativas a partir fontes monitoradas por órgãos públicos e associações profissionais (escolas, pais e meios de comunicação) está associada à redução do consumo de substâncias. Em contrapartida, a informação obtida por fontes informais (irmãos, amigos e *internet*) encoraja o uso de substâncias psicoativas e o consumo mais intenso. Desse modo, ressalta-se a importância da literacia em saúde na promoção de estilos de vida saudáveis entre jovens e da influência das fontes de informação sobre o consumo de substâncias^(25)^.

Não foram identificadas iniciativas da escola ou de serviços de saúde no tocante à criação de espaços de diálogo com os adolescentes sobre o consumo de substâncias psicoativas no ambiente escolar, tampouco sobre qualquer outra temática de interesse dos participantes. Esse cenário se revela propício e desafiador para o planejamento e desenvolvimento de práticas de saúde no ambiente escolar e que agreguem adolescentes, familiares e escola.

Fatores de diversas ordens, como a falta de formação e informação sobre a temática e a sobrecarga de trabalho, desafiam educadores a realizar práticas de prevenção do uso de substâncias psicoativas e promoção da saúde do adolescente no contexto escolar público. Além disso, as representações sociais desses educadores acerca da adolescência e do consumo de substâncias psicoativas entre adolescentes se fundamentam em noções do senso comum, estigmas, preconceitos e julgamentos, o que dificulta o diálogo sobre o tema com os adolescentes e famílias no âmbito escolar. Ainda são escassas as estratégias sistematizadas por educadores para prevenção do consumo de substâncias psicoativas nas escolas, e a maioria daquelas existentes não aborda o tema em um contexto amplo de saúde^(26)^.

As ações de educação e de saúde voltadas para o ambiente escolar, fundamentadas no propósito de uma sociedade sustentável, poderiam considerar os contextos local e regional, de modo a criar condições propícias para que estudantes, familiares, educadores e gestores escolares reflitam criticamente sobre problemas que afetam a humanidade e a vida dos adolescentes, ressignificando conceitos e atitudes socioambientais e repensando estilos de vida. Através de estratégias participativas, horizontalizadas e intersetoriais, é possível fortalecer o compromisso e a execução de comportamentos sustentáveis, além de criar uma cultura de responsabilidade ambiental na escola e na comunidade. As novas demandas mundiais requerem ações globais, sob uma ótica de resolução local, em consonância com agendas mundiais, como a dos ODS^(27)^.

O uso de substâncias psicoativas se revela como um problema global interligado em vários aspectos do desenvolvimento sustentável. Sua análise e consequente resposta, através dos ODS, denotam essa interação, transversalizando a natureza e a dinâmica do problema nos âmbitos individual e coletivo. Programas voltados à prevenção, tratamento, cuidado, reabilitação e reinserção social desempenham papel essencial, e programas baseados em evidências científicas podem reduzir as repercussões negativas do uso de drogas^(28)^.

As estratégias de promoção da saúde e prevenção do uso de substâncias psicoativas a serem planejadas e desenvolvidas no ambiente escolar podem promover espaços de diálogo sobre início precoce do consumo, incentivo familiar ao uso, publicidade e propaganda de substâncias, disponibilidade e acesso. É premente que tais estratégias fortaleçam a colaboração intersetorial, de modo a proteger adolescentes do consumo de substâncias psicoativas e dos danos relacionados^(29)^.

Ressalta-se, ainda, o papel estratégico da Atenção Primária à Saúde no fortalecimento das políticas públicas e no uso de tecnologias do cuidado na atenção humanizada e integral à saúde do adolescente e sua família, fundamentado no reconhecimento desses como sujeitos de direitos. A nível coletivo, estratégias de promoção da saúde emergem como oportunidade de articular práticas educativas em saúde, com vistas a escolhas saudáveis e desenvolvimento da autonomia e do autocuidado no que diz respeito à prevenção e ao consumo de substâncias psicoativas^(20)^.

### Limitações do estudo

Entre as limitações da pesquisa, destacam-se: a realização de apenas uma sessão de grupo focal, havendo a necessidade de aprofundar as informações em outros encontros; a condução do estudo dentro do ambiente escolar, existindo a possibilidade de interferências desse espaço nas respostas dos participantes; e a inclusão apenas dos adolescentes matriculados no 8º ano do ensino fundamental, período matutino. A utilização do grupo focal como técnica de coleta de dados também pode ter inibido alguns participantes em expor opiniões, especialmente nos casos de discordância das ideias de outros membros do grupo.

### Contribuições para as áreas da saúde e enfermagem

O estudo amplia e aprofunda a literatura científica sobre a percepção de adolescentes acerca do uso de substâncias psicoativas, evidenciando a necessidade de potencialização do ambiente escolar como espaço de desenvolvimento de ações de promoção da saúde mental, prevenção do uso de substâncias e educação em saúde. O consumo de substâncias psicoativas é uma barreira para o alcance dos ODS. Nessa perspectiva, profissionais de saúde e enfermagem despontam com responsabilidade e compromisso ímpar no processo de transformação social, participando do planejamento e implementação de políticas e práticas de promoção da saúde e prevenção do uso de substâncias psicoativas, com caráter interdisciplinar, interprofissional e intersetorial, que tenham como alvo os diversos atores que permeiam o cenário escolar.

## CONSIDERAÇÕES FINAIS

As percepções dos adolescentes sobre o uso de substâncias psicoativas revelaram julgamentos, dúvidas e lacunas de conhecimento. O cotidiano dos participantes se mostrou permeado pela maior aceitação de substâncias químicas lícitas, fácil acesso e consumo no território, inclusive no ambiente escolar. Faz-se necessário potencializar o ambiente escolar com ações participativas, horizontais e intersetoriais de promoção da saúde, educação em saúde e prevenção do uso de substâncias psicoativas que incluam estudantes, familiares e escola, visando à reflexão sobre uso de drogas e estilos de vida, e à ressignificação de conceitos e atitudes.

## Data Availability

https://doi.org/10.48331/scielodata.LRC0JH
